# Moiré‐Induced Symmetry Breaking of Charge Order in van der Waals Heterostructures

**DOI:** 10.1002/adma.74730

**Published:** 2026-08-28

**Authors:** Sandra Sajan, Laura Pätzold, Tarushi Agarwal, Clara Pfister, Haojie Guo, Sisheng Duan, P. V. Sruthibhai, Mariana Rossi, Maria N. Gastiasoro, Sara Barja, Ravi P. Singh, Tim Wehling, Miguel M. Ugeda

**Affiliations:** ^1^ Donostia International Physics Center (DIPC) San Sebastián Spain; ^2^ I. Institute of Theoretical Physics University of Hamburg Hamburg Germany; ^3^ Department of Physics Indian Institute of Science Education and Research Bhopal Bhopal India; ^4^ Centro de Física de Materiales (CSIC‐UPV/EHU) San Sebastián Spain; ^5^ Max Planck Institute For the Structure and Dynamics of Matter Hamburg Germany; ^6^ Yusuf Hamied Department of Chemistry University of Cambridge Cambridge UK; ^7^ Dept. Polymers and Advanced Materials University of the Basque Country (UPV/EHU) San Sebastián Spain; ^8^ Ikerbasque, Basque Foundation for Science Bilbao Spain; ^9^ The Hamburg Centre For Ultrafast Imaging Hamburg Germany

**Keywords:** 2D materials, charge density wave, condensed matter physics, misfit layered compounds, pairing, scanning tunneling microscope, stacking, superconductivity, symmetry breaking

## Abstract

Layered materials that stack different lattice symmetries are rare in nature. Misfit layered chalcogenides, which combine square and hexagonal lattices of rocksalt monochalcogenides and transition‐metal dichalcogenides, provide a platform to explore how incommensurability and explicit symmetry breaking impact collective electronic phases. Here we use low‐temperature scanning tunneling microscopy/spectroscopy to probe the misfit compounds (MS)_1+δ_TaS_2_ with M = Pb, Sn and track how the misfit interface reshapes the electronic ground state of the embedded 1H‐TaS_2_ monolayers. High‐resolution STM imaging and Fourier analysis reveal that the charge‐density wave (CDW) is incommensurate and exhibits spatially inhomogeneous, short‐range correlations. Strikingly, the CDW exhibits a pronounced and anisotropic response to the uniaxial moiré potential imposed by the misfit layer: its coherence lengths and ordering wavevectors become inequivalent, demonstrating a strong nonlinear coupling between the intrinsic CDW instability and the symmetry‐breaking moiré field. First‐principles‐informed multiscale modeling suggests that this reorganization arises from the combined effect of interlayer charge transfer and the spatially anisotropic energy landscape introduced by the misfit interface. In contrast, superconductivity is comparatively insensitive to the moiré, revealing a uniform, single full‐gap consistent with s‐wave pairing. Our results establish heterosymmetry stacking as a route to engineer correlated states in van der Waals materials.

## Graphical abstract

A uniaxial moiré potential in misfit (MS)_1+δ_TaS_2_ heterostructures selectively reshape charge order: the embedded 1H‐TaS_2_ layer hosts an incommensurate charge‐density wave, fragmented into nanometer domains and stripped of threefold symmetry. Superconductivity, however, remains uniform and fully gapped, revealing heterosymmetry stacking as a selective handle on correlated quantum phases.

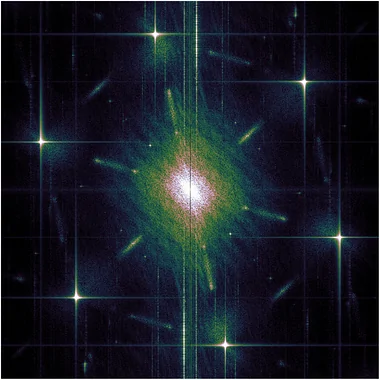

## 1 Introduction

Collective electronic states are highly sensitive to underlying lattice symmetries and superlattice modulations, providing practical knobs to reshape and tune broken‐symmetry phases such as charge order and superconductivity. Two‐dimensional (2D) materials are especially well suited to such control, and recent progress using twisted layers [1, 2, 3] and patterned substrates [4, 5] highlights how engineered periodic potentials can be used to tune correlated phases [6]. Misfit layered compounds, a family of van der Waals materials, emerge as natural platforms to explore symmetry‐breaking effects on collective phases in 2D materials without strongly perturbing their underlying electronic structure. In their simplest stoichiometry, (MX)_1+δ_(TX_2_), these layered materials combine alternating stacks of transition metal dichalcogenide monolayers (TX_2_, with T = Ti, V, Nb, Ta, Mo, W and X = S, Se) and monochalcogenide bilayers (MX, M = Sn, Pb, Bi, and Sb), the latter acting as electronic decouplers [7, 8, 9]. While the presence of charge‐density wave (CDW) [10, 11] and superconductivity [10, 12, 13, 14, 15, 16, 17] is established in these materials, the microscopic mechanisms and key degrees of freedom governing these ordered states remain unknown.

From a symmetry perspective, misfit materials hold the unique property of combining lattices with different symmetries. The MX layers exhibit a rocksalt, square lattice that, upon stacking, lowers the threefold rotational symmetry (*C_3_*) of the 1H‐TaS_2_ layer (point group *D_3h_*). As a result, the square–hexagonal interface generates a uniaxial moiré superlattice potential that acts as an explicit symmetry‐breaking field, capable of lifting the degeneracy between symmetry‐related ordering wavevectors in the TMD layer. Therefore, misfit compounds offer a unique opportunity to directly determine the barely explored impact of such symmetry reduction on delicate collective phases in two dimensions.

Here we report measurements of the electronic structure of two prototypical misfit compounds, (PbS)_1.13_TaS_2_ and (SnS)_1.15_TaS_2_, using high‐resolution low‐temperature scanning tunneling microscopy and spectroscopy (STM/STS) and noncontact atomic force microscopy (nc‐AFM). We find that the MX‐TaS_2_ interface generates a uniaxial (1D) moiré that lifts the threefold degeneracy of the CDW wavevectors of the TMD monolayer, yielding an incommensurate CDW with spatially inhomogeneous, short‐range correlations. Despite the incommensurate order, the wavevector intensity exhibits maxima at rational Bragg (***Q***_B_) positions at $\frac{1}{2}$ ***Q***_B_ for one component, and $\frac{3}{8}$ ***Q***_B_ and its equivalent $\frac{5}{8}$ ***Q***_B_ =−$\frac{3}{8}$ ***Q***_B_ + ***Q***_B_ for the other two components. First‐principles‐based multiscale modelling supports a mechanism behind the observed CDW state, based on the interplay between interlayer charge transfer and the potential‐energy modulation imposed by the misfit moiré. The model reproduces the qualitative tendency toward broadened, anisotropic CDW structure factors, although it does not quantitatively account for the distinct experimental maxima at specific wavevectors. In contrast, STS measurements at 0.34 K reveal a uniform single, full superconducting gap in the TaS_2_ layers consistent with isotropic *s*‐wave pairing, alongside proximitized gaps of slightly smaller magnitude in the monochalcogenide layers, evidencing a highly transparent interface and insensitivity to the moiré potential.

## 2 Results

Our experiments were carried out on high‐quality crystals of (MS)_1+δ_TaS_2_ with M = Pb, Sn (see methods and Supporting Information, section S1, Figures S1–S3). These layered compounds comprise alternating 1H‐TaS_2_ monolayers and rocksalt MS(001) bilayers with a lattice mismatch along a high‐symmetry axis. As illustrated in the sketch of Figure 1a, the square MS lattice lies epitaxially on the hexagonal TaS_2_ lattice such that one MS in‐plane axis aligns with a close‐packed direction of TaS_2_, yielding a commensurate registry along *y*. Along the orthogonal *x* direction, however, the intrinsic periodicities of the square and hexagonal lattices differ (*a*_PbS_ = 5.78 Å, *a*_SnS_ = 5.75 Å, and $a_{{\text{TaS}}_{2}}$ = 3.30 Å), producing a one‐dimensional (1D) incommensurability. This mismatch accumulates laterally to generate a long‐wavelength moiré modulation parallel to *x*, while the layers remain structurally locked along *y*. The impact of this moiré on the properties of the collective electronic states of the TMD layers remains poorly understood, and constitutes the main goal of this work.

**FIGURE 1:**
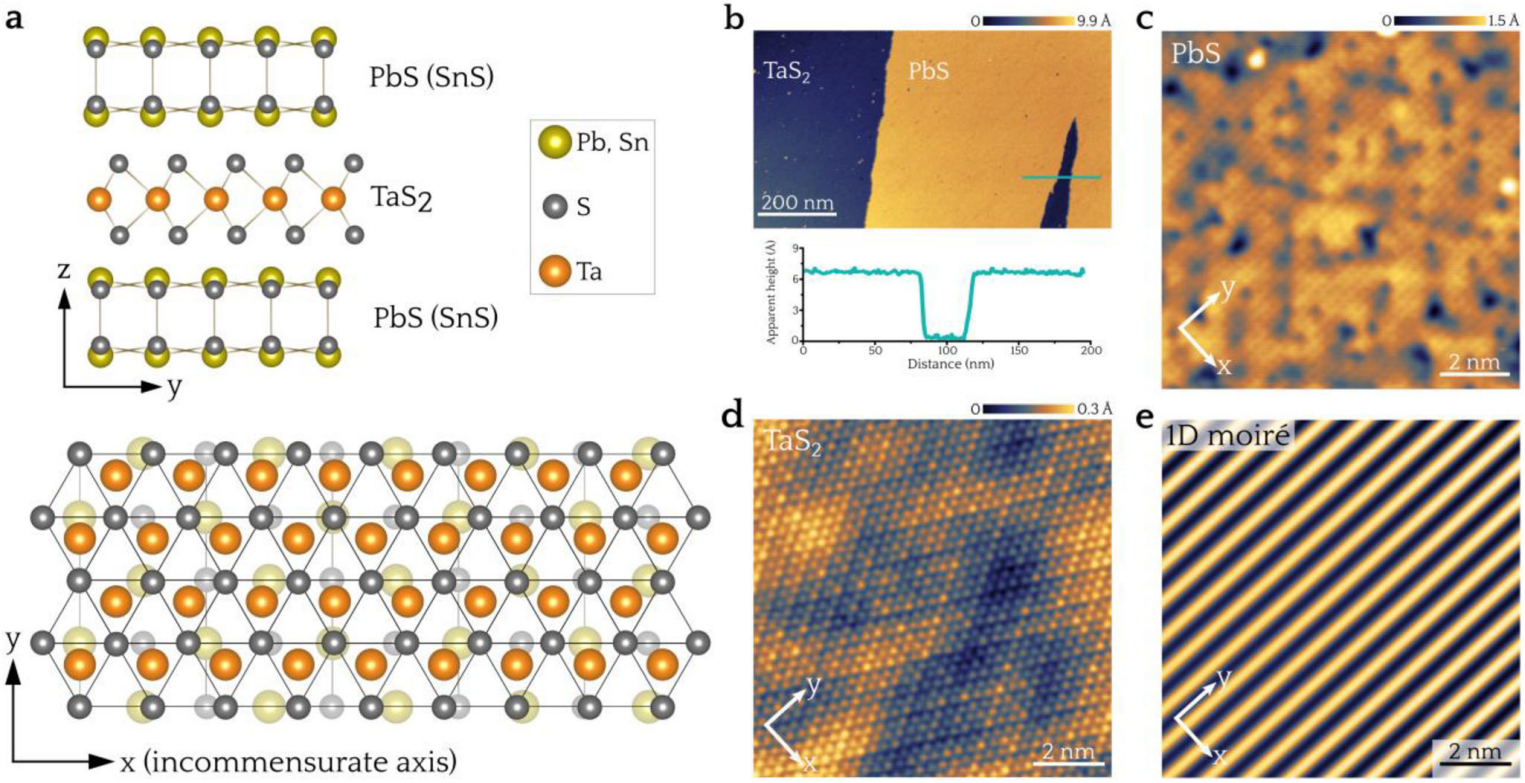
Structure and moiré of monochalcogenide–TaS_2_ misfit compounds. (a) Side and top view sketches of (MS)_1+δ_TaS_2_ (M = Pb, Sn). (b) Large‐scale STM topography showing the PbS and TaS_2_ terrace layers (*V*_s_ = 1 V, *I* = 100 pA, *T* = 4.2 K). The height profile below is taken along the green line in the image. (c,d) Atomically resolved STM images of PbS (*V*_s_ = 0.3 V, *I* = 260 pA, *T* = 0.34 K) and TaS_2_ (*V*_s_ = 0.1 V, *I* = 320 pA, *T* = 4.2 K), respectively. (e) Wavefront of the 1D moiré formed between the PbS and TaS_2_ crystal lattices extracted by taking the inverse FFT of the image in (d).

Figure 1b illustrates the typical morphology of our samples—(PbS)_1.13_TaS_2_ in this case—after exfoliation (see methods), showing terraces exposing adjacent PbS and TaS_2_​ terminations with a step height of ∼7 Å. Figure 1c,d show atomically resolved STM images of the PbS and TaS_2_ layers, respectively. On PbS, the STM images show a square lattice where both atomic species appear with comparable topographic contrast. Atomic defects, presumably vacancies, are also present in the PbS surface. On TaS_2_, STM images exhibit the trigonal atomic registry, upon which an apparently incommensurate superlattice is overlaid, reminiscent of a CDW modulation. We found the same morphology in the SnS and TaS_2_ layers of (SnS)_1.15_TaS_2_ (section S2, Figure S4). Notably, this incommensurate superlattice clearly departs from the CDW orders previously reported for isolated 1H‐TaS_2_ monolayers, close to a 3 × 3 or a 2 × 2 periodicity, depending on doping [18, 19, 20]. This observation is also in contrast to the periodicity found in the isostructural analogue (SnS)_1.15_TaS_2_, where a similar CDW order close to a 3 × 3 has been reported [10, 11]. This constitutes a first indication that the MS/TaS_2_ misfit environment stabilizes a distinct, incommensurate charge modulation.

Although the presence of the 1D moiré arising from the square and hexagonal lattices is not apparent in the topography images, especially in (PbS)_1.13_TaS_2_ (section S2 for the (SnS)_1.15_TaS_2_ case), its periodicity and orientation can be extracted from the Fourier‐transform (FFT) analysis. Figure 1e shows the real‐space modulation of the moiré with respect to the crystal orientations of PbS and 1H‐TaS_2_ in the regions in Figure 1c,d. Along the incommensurate *x* direction, the difference between the first reciprocal‐lattice vectors of the two layers defines $|q_{\mathrm{moire}}\left|=\right|G_{{\mathrm{TaS}}_{2},x}-G_{\mathrm{MS},x}|\approx$ 0.82 Å^−1^, corresponding to a short‐period modulation of $\lambda_{\mathrm{moir}\overset{´}{e}}$ = 7.69 Å, or 7.74 Å in (SnS)_1.15_TaS_2_. The wavevector ***q**_moire_* lies along MS [100] and TaS_2_ [10$\bar{1}$]. This short‐period modulation is the third harmonic of a 2.3 nm moiré supercell in (PbS)_1.13_TaS_2_, defined by the near‐commensurate matching condition $7a_{{\mathrm{TaS}}_{2\;}}≅4a_{\mathrm{MS}}$. Thus, 2.3 nm is the fundamental registry‐repeat period, while 7.7 Å is the dominant short‐period Fourier component. The 1D superlattice breaks the threefold rotational symmetry of 1H‐TaS_2_, reducing it to a twofold rotational symmetry aligned with the misfit *y* axis.

To study the microscopic properties of the electronically ordered phases in these misfit compounds, we first investigated the electronic structure of both surface terminations (PbS and TaS_2_) using low‐temperature STS. Figure 2a shows two typical d*I*/d*V* spectra (d*I*/d*V* ∝ local density of states) acquired consecutively on two alternating terraces of PbS and TaS_2_ (section S3 for the (SnS)_1.15_TaS_2_ data (Figure S5)). The observed electronic structure of the TaS_2_ monolayer is dominated by a broad resonance located at +0.9 V with a significantly reduced density of states (DOS) at lower energies down to −1 V. The DOS lineshape is reminiscent of previous STS measurements on TaS_2_ monolayers on graphene substrates [20, 21], where mutual electronic coupling is minimal [22]. We attribute this resonance to *d*‐states in the flat region of the Ta‐derived band near $\bar{\Gamma }$, similar to other metallic TMD monolayers [23, 24]. These observations are consistent with recent ARPES measurements [10, 15, 16] on (PbS)_1.13_TaS_2_, which reveal a negligible interlayer hybridization and a largely rigid, electron‐doped TaS_2_ band structure (∼0.2 electron per Ta) [15]. In the PbS bilayer, the electronic structure shows a reduced DOS in the broad range from +0.1 to −1 V. This agrees qualitatively with a predicted gap of ∼0.2 eV for isolated PbS together with a ∼1 eV depletion of the DOS around *E*_F_ due to a steep band dispersion around $\bar{L}$ [25]. Experimentally, however, the PbS bilayer shows a reduced but finite DOS in the STS measurements, a behavior also expected in the PbS layers when embedded in the misfit compound that implies a charge transfer to the TaS_2_ layer [25].

**FIGURE 2:**
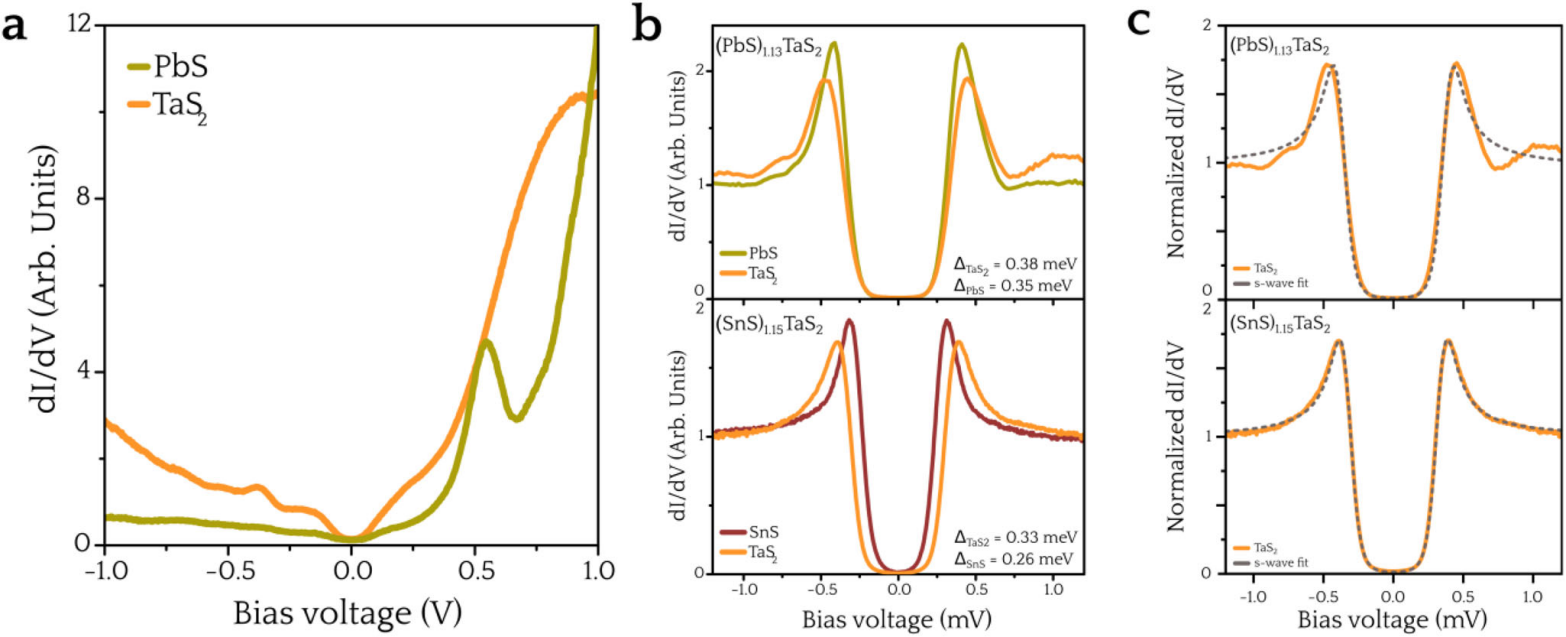
Electronic structure and superconductivity. (a) Wide‐bias d*I*/d*V* spectra consecutively acquired on TaS_2_ (orange) and PbS (green) at *T* = 4.2 K. (b) Low‐bias d*I*/d*V* spectra consecutively acquired on TaS_2_ and PbS terminations of (PbS)_1.13_TaS_2_, with $\Delta_{{\mathrm{TaS}}_{2}}=\left(0.38\pm 0.04\right)\;\mathrm{meV},\Gamma_{{\mathrm{TaS}}_{2}}=\left(0.020\pm 0.004\right)\;\mathrm{meV},\Delta_{\mathrm{PbS}}=\left(0.35\pm 0.04\right)\;\mathrm{meV},\Gamma_{\mathrm{PbS}}=\left(0.020\pm 0.006\right)\;\mathrm{meV}$), and on the TaS_2_ and SnS terminations of (SnS)_1.15_ TaS_2_ ($\Delta_{{\mathrm{TaS}}_{2}}=\left(0.33\pm 0.03\right)\;\mathrm{meV},\Gamma_{{\mathrm{TaS}}_{2}}=\left(0.020\pm 0.002\right)\;\mathrm{meV},\Delta_{\mathrm{SnS}}=\left(0.260\pm 0.003\right)\;\mathrm{meV},\Gamma_{\mathrm{SnS}}=\left(0.010\pm 0.003\right)\;\mathrm{meV}$). All spectra show a single, fully open superconducting gap (T = 0.34 K). (c) BCS s‐wave fit to the d*I*/d*V* spectra on the TaS_2_ layers shown in (b).

The metallic character of both PbS and 1H‐TaS_2_ layers enables the development of superconductivity in each layer, which we probed using high‐resolution STS at 0.34 K. Although the presence of superconductivity in the Pb‐ and Sn‐based (MS)_1+δ_TaS_2_ compounds is established primarily via mesoscopic techniques [10, 12, 14, 15, 17, 26] (section S1, Figures S2, S3), their microscopic, local properties remain scarcely explored [11]. Previous transport measurements in these and related compounds indicate a robust 2D Ising character with a large, anisotropic *H_C2_* consistent with 2D Tinkham scaling [10, 14, 15, 17, 26, 27]. Figure 2b shows four representative d*I*/d*V* spectra acquired consecutively on the TaS_2_ monolayer and the corresponding monochalcogenide layer in the (PbS)_1.13_TaS_2_ and (SnS)_1.15_TaS_2_ compounds. As can be seen, all layers display a hard gap with coherence peaks characteristic of superconductivity. In TaS_2_ layers, we consistently observe for both compounds a single gap that is well described by an isotropic BCS fit, as shown in Figure 2c. The BCS gap values—Δ_TaS2_ = (0.38 ± 0.03) meV in (PbS)_1.13_TaS_2_ and Δ_TaS2_ = (0.33 ± 0.03) meV in (SnS)_1.15_TaS_2_ – together with the critical temperatures of these compounds (*T*_C_ = 2.7–3 K), yield 2Δ/*k*_B_ *T*_C_ ratios of 2.6–3.3, which lie slightly below the weak‐coupling BCS value (3.53).

In PbS and SnS, the superconducting gaps are slightly smaller than those in TaS_2_, consistent with a proximitized superconductivity in the monochalcogenide layers. In both cases, the gaps are well fit by a BCS form (section S4, Figure S6). Furthermore, both the monochalcogenide and dichalcogenide layers in (PbS)_1.13_TaS_2_ show similar responses to out‐of‐plane magnetic fields (*H* ^⟂^), with a common critical value of *H* ^⟂^_C2_ ≃ 0.2 T (section S5 (Figure S7)). The large gap ratios *r* = Δ_PbS‐SnS_/Δ_TaS2_ near unity point to a highly transparent interface for superconductivity [28, 29]. Lastly, our spatially resolved STS data show uniform gaps on both the TMD and monochalcogenide layers, which indicates efficient superconducting proximity coupling across a highly transparent interface, likely mediated by pair tunneling/Andreev processes between the layers. No moiré‐induced modulation of the superconducting gap is resolved within the spatial and energy resolution of the present measurements (section S6, Figure S8). Overall, our observation of a single, BCS‐like superconducting gap in the monolayers of TaS_2_ and PbS (SnS) contrasts with reports of unconventional pairing phenomenology, including nodal [20] and mixed singlet–triplet [11] gap structures.

We next focus on probing the CDW order in the 1H‐TaS_2_ monolayers. As mentioned above, the STM images of TaS_2_ show a 2D superlattice that appears incommensurate with the underlying lattice (Figure 1d). Although compatible with CDW order, this incommensuration strikingly contrasts with the nearly commensurate CDW order reported for 1H‐TaS_2_ in bulk crystals and down to the monolayer limit, including in misfit compounds [10, 11, 27]. To better understand and interpret the observed incommensurate 2D superlattice, we perform Fourier analysis of atomically resolved STM topographies of TaS_2_ across extended fields of view. Figure 3a shows a large‐scale STM image of TaS_2_ in (PbS)_1.13_TaS_2_ with atomic resolution, whose FFT is shown in Figure 3b. Apart from the Bragg peaks ***Q***_B_, the FFT reveals three elongated features labelled as ***q*** *_i_* (*i* = 1,2,3) along the Bragg directions and a sharp peak at ***q***_moiré_ = (0.84 ± 0.01) Å^−1^ · ***q*** *_x_*, where ***q*** *_x_* is the unit vector in *x*‐direction of reciprocal space. The latter corresponds to a 1D periodicity in real space of *λ* = (7.5 ± 0.1) Å, which we attribute to the striped moiré with wavelength *λ*_moiré_ formed by the square and hexagonal lattices. The elongated features ***q**_i_*, aligned with the crystal TaS_2_ lattice, exhibit two distinct internal structures. The ***q***_1_ feature, orthogonal to ***q***_moiré_, shows intensity along an extended region around $\frac{1}{2}\;$ ***Q***_B_ (2 × 2 in real space), where the intensity is maximum (Figure 3c). Instead, ***q***_2_ and ***q***_3_, rotated 30° with respect to ***q***_moiré_, share an equal distribution of intensities with two maxima at $\frac{3}{8}$ ***Q***_B_ and $\frac{5}{8}$ ***Q***_B_ and a slow decay toward $\frac{1}{2}$ ***Q***_B_—the $\bar{M}$ point (Figure 3c). The shape and maxima of these features are independent of the STM imaging bias voltage (Figure 3d), which rules them out as quasiparticle interference features and are, instead, fully compatible with CDW order. The intensity profiles along ***q***_1_ and ***q***_2,3_ shown in Figure 3c reveal nearly equal extents of the lines around $\bar{M}$ despite the maxima occurring at different wavevectors. Remarkably, no noticeable intensity has been found close to $\frac{1}{3}$ ***Q***_B_, which corresponds to the usual near 3 × 3 CDW periodicity in real space for undoped TaS_2_. We found similar behavior of the CDW phase in the TaS_2_ layers of the (SnS)_1.15_TaS_2_ compound (section S7, Figure S9). Lastly, we emphasize that differential conductance maps show the same features as in the topographic FFTs, confirming their presence in the local DOS. Instead, our nc‐AFM images do not reveal any significant structural distortion associated with the modulations, supporting their assignment to electronic CDW order rather than structural effects (section S7, Figures S10).

**FIGURE 3:**
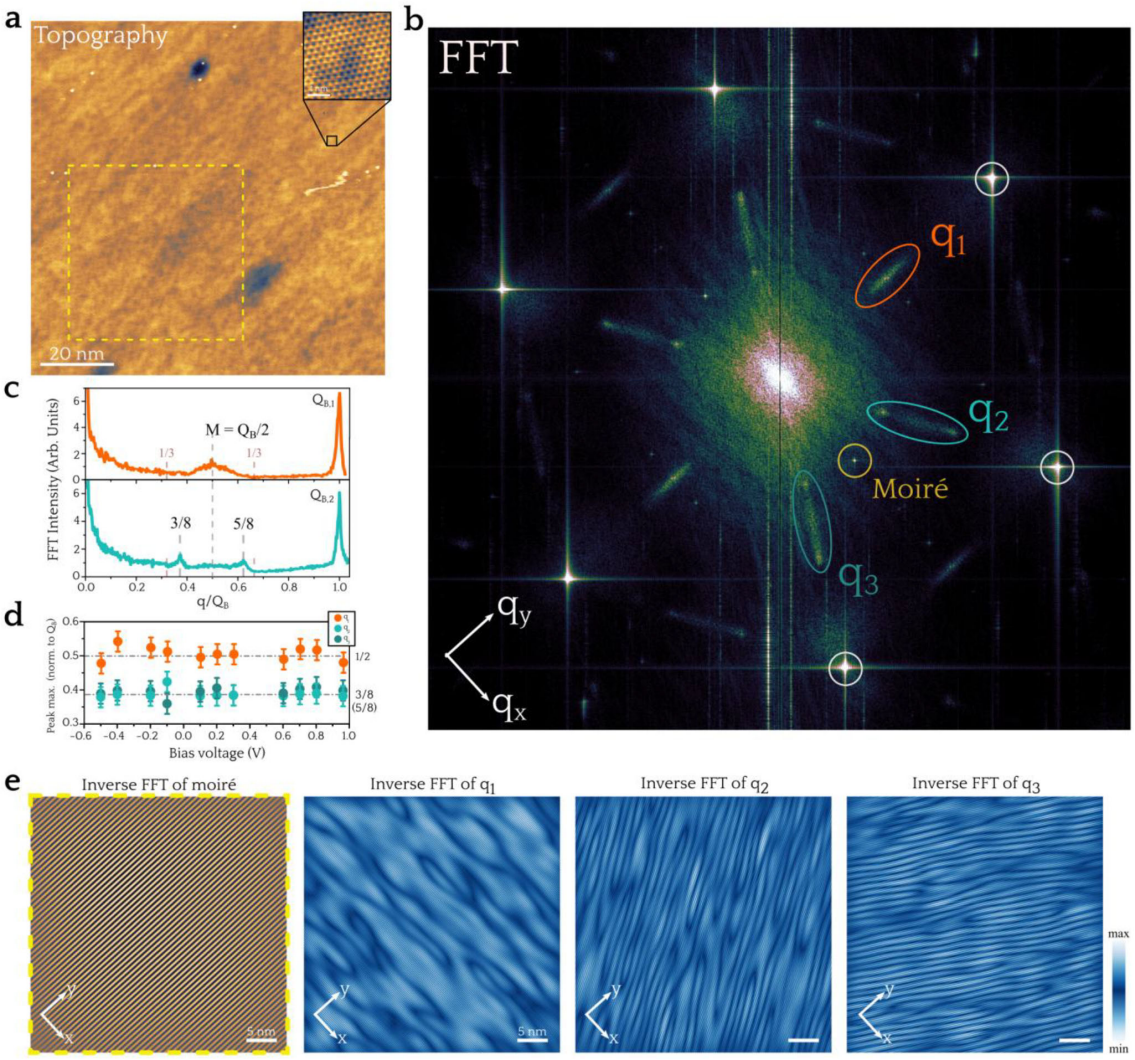
Broken lattice degeneracy in the CDW of (PbS)_1.13_TaS_2_. (a) Large‐scale STM image with atomic resolution in 1H‐TaS_2_ (*V*_s_ = 0.6 V, *I* = 500 pA, *T* = 4.2 K) and (b) its corresponding Fourier transform (FFT). (c) Intensity profiles of the FFT along $\Gamma -Q_{B,1}$ (orange) and $\Gamma -Q_{B,2}$ (cyan). (d) Energy position of the maxima of the CDW features in the FFT as a function of bias voltage. (e) Inverse FFT of the moiré peak (left panel) and the ***q*** *_i_* elongated features (blue) of the region boxed in yellow in (a).

To gain further insight into the spatial organization of the CDW order in the TaS_2_ layers, we isolated each ***q*** *_i_* feature in reciprocal space and inverse Fourier transformed the corresponding masked complex Fourier coefficients. Figure 3e shows the resulting real‐space maps within the region boxed in Figure 3a, together with the 1D‐moiré oscillation as a reference. In the maps, light‐blue regions indicate larger CDW amplitudes, whereas dark regions correspond to regions of reduced amplitude. As seen, all three ***q*** *_i_* components are inhomogeneous and exhibit short‐range CDW correlations over nanometer length scales. The reciprocal‐space broadening provides an order‐of‐magnitude estimate of the corresponding correlation lengths through Δ ***q*** *_i_* ∼ 2π/*L_i_*, but may also reflect a distribution of local wavevectors or phase disorder. For ***q***_2_ and ***q***_3_, most of the intensity is distributed continuously between two maxima at $\frac{3}{8}$ ***Q***_B_ and $\frac{5}{8}$ ***Q***_B_, defining a reciprocal‐space width of Δ ***q**_2,3_* = (0.57 ± 0.02) Å^−1^ ≃ $\frac{1}{4}$ ***Q***_B_. For ***q***_1_, the signal is spread around a long arc of width Δ ***q***_1, FWHM_ = (0.28 ± 0.08) Å^−1^ centered on $\bar{M}$, indicating a wide distribution of characteristic length scales. An autocorrelation analysis of the filtered components yields characteristic decay lengths of $\tau_{q_{1}}\approx 1\;\mathrm{nm}$, $\tau_{q_{2}}\approx 1.8\;\mathrm{nm}$ and $\tau_{q_{3}}\approx 2.6\;\mathrm{nm}$, confirming that the CDW correlations are short ranged and anisotropic. These observations demonstrate the full incommensuration of the CDW order in the 1H‐TaS_2_ monolayers of the (MS)_1+δ_TaS_2_ (M = Pb, Sn) compounds.

These findings show the incommensurate nature of the CDW order in the 1H‐TaS_2_ monolayers of misfit compounds and explain why previous resistivity and Raman measurements show no CDW‐related anomalies [15, 16, 17, 27], while ARPES detects no band folding, consistent with suppression of a commensurate CDW. This moiré‐induced exotic CDW order departs from the nearly commensurate and *C_3_*‐symmetric behavior reported for this TMD metal.

To better understand and interpret our experimental findings on the threefold degeneracy breaking in the CDW order of the monolayer TMD, we performed first‐principles based simulations of the CDW thermodynamics using monolayer 1H‐TaS_2_ as a minimal model system. Employing downfolding‐based structural relaxations and replica‐exchange molecular dynamics (REMD), we simulated an 18 × 18 supercell. Starting from pristine 1H‐TaS_2_, the structure relaxes into the 3 × 3 CDW reconstruction for the undoped system (see section S8, Figure S11 for the model relaxation). For comparison with the experiments, we analyze the structure factors corresponding to the relaxed structures obtained via REMD simulations in Figure 4 (see section S8 for its definition), corresponding to the Fourier‐transformed STM topography. In the pristine case in Figure 4a, the dominant spectral weight appears at $\frac{1}{3}$ ***Q***_B_, corresponding to the 3 × 3 CDW reconstruction. Upon electron doping, our model relaxations reveal multiple competing commensurate and incommensurate CDW states (see phase diagram in section S9, Figure S12), with the dominant CDW instability vector shifting progressively toward the Brillouin zone edge (see the evolution of structure factors with increasing doping in section S9, Figure S13). For an electron doping of 0.2e per TaS_2_ unit cell (corresponding to the determined doping of a 1H‐TaS_2_ layer integrated in the Pb‐based misfit compound via ARPES [15]), the thermodynamically relevant CDW peaks are shown in Figure 4c. Rather than sharp peaks as in the pristine case, we observe an elongated region of enhanced intensity centered around $\frac{4}{9}$ ***Q***_B_ that extends toward the zone boundary at $\frac{1}{2}$ ***Q***_B_. These broadened intensity features are consistent with the short CDW coherence lengths observed experimentally. Doping thus explains the shift of the CDW vectors away from $\frac{1}{3}$ ***Q***_B_ [7, 27, 30] as well as the short coherence lengths, though not the threefold CDW degeneracy breaking seen in the experiments.

**FIGURE 4:**
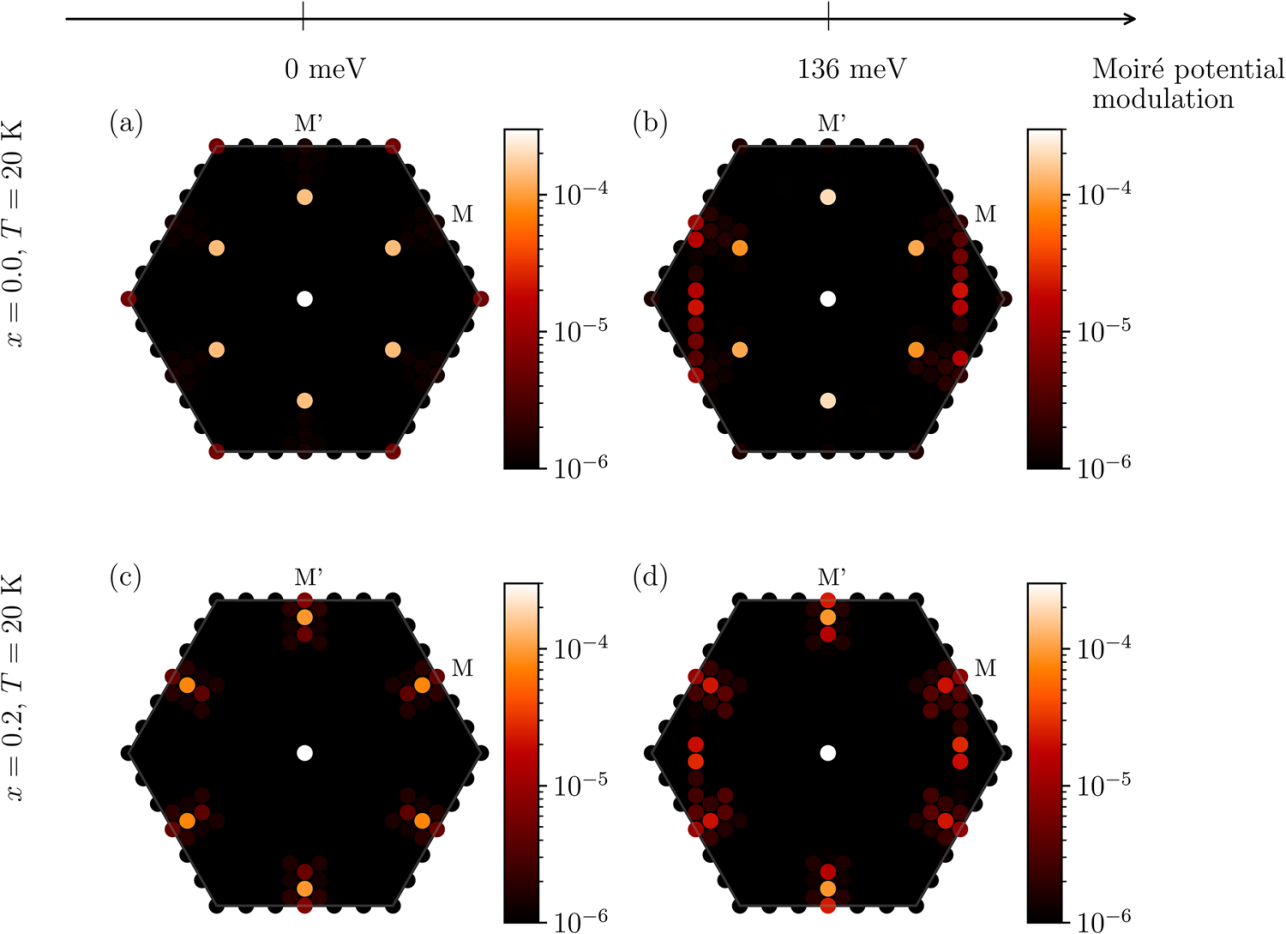
Influence of charge transfer and moiré potential on the CDW state. Structure factors from REMD simulations for the replica at *T* = 20 K for an 18 × 18 1H‐TaS_2_ supercell. Charge‐neutral (*x* = 0.0, (a,b)) and electron‐doped 1H‐TaS_2_ (+0.2e per 1H‐TaS_2_ unit cell, *x* = 0.2, (c,d)) are shown without (a,c) and with (b,d) a 136 meV moiré potential modulation. The undoped supercell (a) verifies the expected 3 × 3 CDW, while electron doping shifts the system into a 2 × 2‐like incommensurate CDW configuration in (c), in line with the short CDW coherence lengths in the experiment. The simulation of interlayer hybridization via a vertically striped electronic potential acting on the downfolded Hamiltonian enforces symmetry breaking as well as a smeared‐out moiré contribution around ***q***_moiré_ in the undoped system (b). Combining the doping‐induced shift towards $M=\frac{1}{2}$ ***Q***_B_ as well as hybridization effects in (d), the central experimental CDW signatures are reproduced qualitatively.

A key distinction between the misfit compound and the monolayer model considered so far is the presence of the one‐dimensional moiré superstructure. To assess its impact on the emergent CDW state, we incorporate hybridization effects between 1H‐TaS_2_ and the underlying MS layer through a spatially modulated electronic potential acting on the Wannier orbitals of the downfolded Hamiltonian. Specifically, we impose a vertically striped pattern with moiré periodicity and an amplitude of 136 meV, which is in the typical order of magnitude for interlayer interactions in transition‐metal dichalcogenide based 2D materials [31] (see Methods section and Figure 5 for more details). For the undoped model (Figure 4b), the moiré potential lifts the degeneracy of the $\frac{1}{3}$ ***Q***_B_ peaks through a change in their intensities (see the evolution of structure factors with increasing moiré potential amplitude in section S10, Figure S14). In addition, a smeared contribution emerges around ***q***_moiré_. Doping enhances the response of the CDW state to the moiré potential. The doped system with moiré potential (Figure 4d) shows a stronger distinction between those CDW Fourier spots orthogonal and non‐orthogonal to ***q***_moiré_ and reveals distinct spreads in ***k***‐space between these two sets of CDW peaks.

**FIGURE 5:**
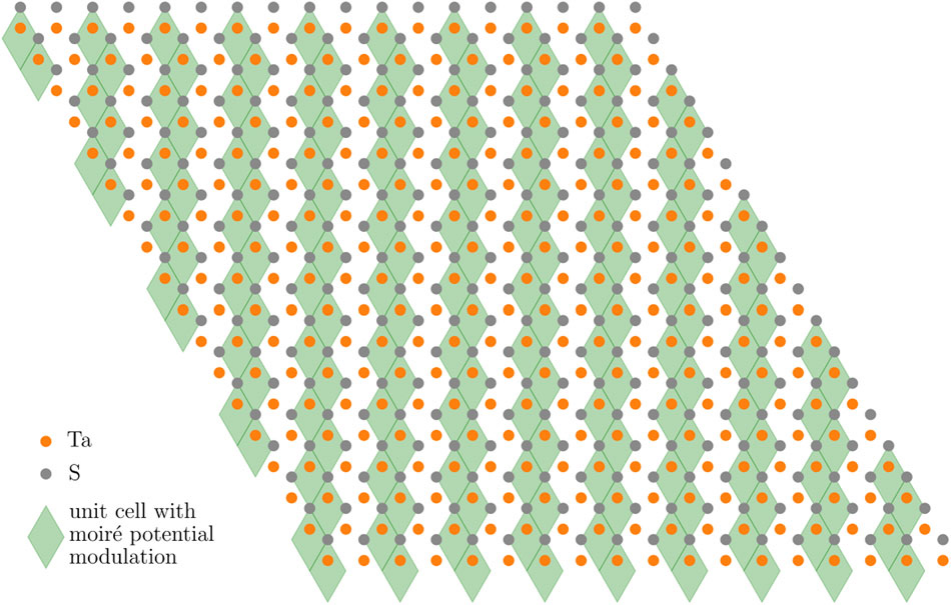
Vertically striped moiré pattern to model the potential modulation of the underlying square lattice. Top view of an exemplary 18 × 18 supercell of 1H‐TaS_2_ in the high‐temperature symmetric phase. To model the resulting 1D moiré pattern due to the hybridization with an underlying MS layer, a spatially modulated electronic potential is incorporated acting on the Ta‐centered Wannier orbitals of the downfolded Hamiltonian. We impose a vertically striped pattern of alternating potential influence onto the 1H‐TaS_2_ layer, with each unit cell marked in green where the MS moiré potential is considered as a constant energy offset on the on‐site terms of the downfolded Hamiltonian.

Our minimal model captures essential ingredients of the misfit heterostructure while deliberately reducing its microscopic complexity. As expected for such a simplified description, certain details differ from experiment: in the model, the moiré‐induced symmetry breaking manifests as variations in peak intensities and ***k***‐space extent rather than as distinct maxima at shifted wave vectors. Notwithstanding these differences, the calculations show that the combination of a doping‐induced shift towards a 2 × 2–like instability and an enhanced susceptibility to the moiré modulation (Figure 4d) lead to a markedly strong symmetry breaking of the CDW, the shifts of the CDW maxima toward the Brillouin zone boundary, as well as the reduced CDW coherence at a qualitative level (cf. Figure 3b). For the simulation in Figure 4d, the structure factors associated with select MD replicas at different temperatures are shown in the Supporting Information (see section S11, Figure S15). Although some thermal broadening and enhanced diffuse scattering is visible at higher temperatures, the qualitative characteristics of the symmetry‐broken structure factor remain stable for temperatures up to *T* = 50 K, with the moiré peak persisting up to the highest replica temperature of *T* = 200 K.

## 3 Discussion

Our results reveal a clear hierarchy in the sensitivity of collective electronic phases to the misfit interface. Superconductivity remains fully gapped and well described by an isotropic BCS spectrum in the 1H‐TaS_2_ layers, with slightly reduced proximity‐induced gaps in the adjacent monochalcogenide layers, consistent with a highly transparent interface. Our experiments do not show any signatures of multigap [11, 14, 15] or unconventional pairing [11, 20], and no spatial modulation of the superconducting gap associated with the misfit superlattice is resolved within the present experimental resolution.

In contrast, the charge‐density wave proves to be markedly more susceptible. Although CDW order persists along the reciprocal‐lattice directions defined by the 1H‐TaS_2_ Bragg vectors ***Q***_B_, its internal degrees of freedom are strongly reshaped: the magnitudes of the CDW wave vectors, the CDW coherence lengths, and the relative Fourier intensities along the three inequivalent ***Q***_B_‐directions become markedly unequal. Because the moiré wave vector lies along a distinct reciprocal‐space direction ***q***_moiré_ ∦ ***Q***_B_, this behavior cannot arise from simple locking, but instead reflects nonlinear, anharmonic coupling between the misfit‐induced potential landscape and a near‐degenerate manifold of CDW instabilities in the ***Q***_B_‐directions. First‐principles‐informed modeling shows that interlayer charge transfer shifts the CDW Fourier peaks toward the zone boundary, while the moiré‐modulated interlayer potential lifts the residual three‐fold rotational degeneracy, stabilizing an anisotropically deformed CDW state. These findings demonstrate that CDW formation in 1H‐TaS_2_ is robust in its preferred ordering directions, yet intrinsically soft in its wave‐vector magnitude and coherence.

Our work demonstrates the viability of imposing superlattice potentials on 2D materials to selectively manipulate delicate collective electronic phases while leaving their underlying electronic structure largely intact, as exemplified by the misfit compounds studied here. More broadly, our results suggest that symmetry‐ or lattice‐mismatched stacking can be used to perturb and reshape collective electronic order in layered quantum materials. Many correlated layered materials, including cuprates, Fe‐based superconductors, and kagome metals, display a delicate competition between superconductivity, density‐wave order, nematicity, and other broken‐symmetry phases. The observation that the CDW in 1H‐TaS_2_ can be strongly reconstructed by an adjacent mismatched layer therefore motivates exploring lattice‐mismatched interfaces as a means of tuning such competing phases.

## 4 Methods

### 4.1 Crystal Synthesis

Single crystals were grown by the chemical vapor transport (CVT) method. Prereacted polycrystalline powders were used as the source material for the synthesis of single crystals. The polycrystalline precursors were prepared by thoroughly mixing the constituent elements in their respective stoichiometric ratios and heating the mixtures at 900°C. The resulting prereacted powders were sealed in evacuated quartz ampoules together with transport agents. Iodine served as the transport agent for (PbS)_1.13_TaS_2_, while NH_4_Cl was used for (SnS)_1.15_TaS_2_. The sealed tubes were then placed in a two‐zone tubular furnace with the growth zone temperature set to 850°C for (PbS)_1.13_TaS_2_ and 800°C for (SnS)_1.15_TaS_2_ and maintained for 10 days. Single crystals of few millimeters in size were obtained for both compounds.

### 4.2 STM/STS and Nc‐AFM Measurements

All experiments were performed in two independent ultrahigh vacuum (UHV) systems (Unisoku Co., LTD): (I) an USM‐1300 that operates at temperatures down to 0.34 K and equipped with perpendicular magnetic fields up to 11T and (II) a closed‐cycle cryogen‐free STM/nc‐AFM combined microscope (USM‐1800) operating at 3.8 K. In both systems, single crystals of (PbS)_1.13_TaS_2_ and (SnS)_1.15_TaS_2_ were mechanically exfoliated in UHV conditions. Afterward, the sample was directly transferred to the STM/nc‐AFM stage. Data were acquired at a temperature detailed in each Figure caption. Tunneling spectroscopy (d*I*/d*V*) data were recorded using standard lock‐in amplifier techniques, where a modulation voltage (*V*_a.c_ *_._*) at a frequency of 833 Hz is coupled to the sample bias voltage (*V*_s_). We used typically *V*_a.c._ values of 5–20 µV for d*I*/d*V* spectra within meV range and 1–5 mV for larger energy scales (± 1 V). The energy resolution of the USM‐1300 STM used for the superconducting‐gap measurements is periodically assessed by Josephson spectroscopy at *T* = 0.34 K, yielding *Γ* ≈ 0.10 meV, in agreement with the expected thermal broadening of 3.5*k*_B_ *T* ≈ 0.103 meV; the resolution is therefore thermally limited, with no measurable additional electronic broadening. For constant‐height nc‐AFM imaging (USM 1800), a tuning‐fork‐based qPlus sensor [32] (resonant frequency *f*_0_ ≈ 28.7 kHz; *Q* = 28855) was employed. The microscope was operated in frequency‐shift mode while maintaining a constant oscillation amplitude of 12 Å. Before each experiment, the STM/nc‐AFM tips (made of Pt/Ir) were metallized on single crystals Au(111) or Cu(111) and calibrated against their corresponding Shockley surface state. STM/STS and nc‐AFM data analyzing and rendering were carried out with the WSxM software [33].

### 4.3 Superconducting‐Gap Fitting

To model the DOS (*ρ_s_*) of the superconducting gaps, we employed the expression developed by Dynes:

(1) $$\rho_{s}\left(E\right)=\rho_{s,N}Re\left[\frac{\left|E-i\Gamma \right|}{\sqrt{{\left(E-i\Gamma \right)}^{2}+\Delta^{2}}}\right]$$

where *ρ_s,N_* is the DOS of the sample in the normal state, *Γ* is the quasiparticle lifetime broadening due to all nonthermal sources, and *Δ* is the superconducting gap size. For the sake of simplicity, we considered *ρ_s,N_* as a constant or linear function of energy. Therefore, and assuming an energy‐independent constant DOS of our STM tip, the total differential conductance (d*I*/d*V*) of the sample measured through STS is:

(2) $$dI/dV\left(V\right)\propto \int_{-\infty }^{+\infty }dE\frac{df\left(\varepsilon \right)}{d\varepsilon }{|}_{\varepsilon =E-\mathrm{eV}}\rho_{s}\left(E\right)$$

where *f*(ε) is the Fermi–Dirac distribution that weights for the thermal broadening. The calculated differential conductance was scaled by a linear background, (mV+c), with the slope and offset treated as free fitting parameters to account for the normal‐state conductance background.

### 4.4 Fourier‐Filtering Procedure

Fourier analysis was performed using the WSxM software [33]. For each STM image, the complex two‐dimensional Fourier transform was calculated. For the inverse Fourier‐transform analysis, each CDW component was isolated using multiple elliptical pass masks distributed along the corresponding elongated ***q*** *_i_* feature. The masks were chosen to encompass the full CDW signal while excluding the Bragg peaks, the zero‐frequency background, and the moiré peak. The complete complex Fourier coefficients, including their phase, were retained. Equivalent masks were applied symmetrically around + ***q*** *_i_* and −***q*** *_i_*, yielding a real‐valued filtered image after inverse transformation. The robustness of the reconstruction was tested using hard‐edged rectangular and elliptical masks, as well as smoothly apodized Hann and more strongly apodized Blackman–Harris masks. Comparable spatial distributions and characteristic length scales were obtained in all cases, including upon moderate variations of the mask dimensions. This demonstrates that the conclusions are insensitive to reasonable choices of mask shape, apodization, and size. The corresponding comparison is shown in the Supporting section S12. Lastly, the spatial autocorrelation function of each inverse‐Fourier‐filtered ***q_i_*** ​ component was calculated and its decay envelope fitted with an exponential function.

### 4.5 Density Functional Theory and Downfolding Model

DFT calculations were performed via QUANTUM ESPRESSO [34] using the PBE approximation for the exchange‐correlation potential [35]. For 1H‐TaS_2_, norm‐conserving pseudo‐potentials from the pslibrary database [36, 37] were utilized. Plane waves until an energy cutoff of 100 Ry were included and a Fermi‐Dirac‐type smearing of 0.001 Ry was imposed. We included a vacuum layer of approximately 12 Å above the monolayer and used an 18 × 18 ***k***‐point grid for the unit cell. Relaxation of the unit cell for fixed cell height yielded a lattice constant of 3.34 Å for the undoped model at *x* = 0.0, with (3.35–3.41) Å for the doped models at electron dopings of *x* = 0.05–0.3. The DFPT calculations were also done via QUANTUM ESPRESSO, where we used a 6 × 6 ***q***‐point grid. The generation of maximally localized Wannier functions was performed with the WANNIER90 code [38] as interfaced via QUANTUM ESPRESSO, with three energy bands around the Fermi energy transformed into the Wannier basis starting from orbitals of Ta *d_z_^2^*‐, *d_x_^2^_−y_^2^*, and *d_xy_*‐character. The electron‐phonon coupling was determined via the EPW code [39]. The relaxations on large supercells (18 × 18) were facilitated by the “model III” downfolding scheme [40], which has been implemented in the ELPHMOD package [41]. Their equivalence to DFT had been established for a variety of metallic TMD materials [40]. For the model setup, the same parameters as in DFT were used except for the ***k***‐point grid, which corresponds to a Γ‐only calculation on the 18 × 18 supercell.

### 4.6 Moiré Potential Modulation

In our model, hybridization effects between 1H‐TaS_2_ and the underlying MS layer are incorporated via a spatially modulated electronic potential acting on the Ta‐centered Wannier orbitals of the downfolded Hamiltonian. To model the 1D moiré pattern of the square lattice, we impose a vertically striped pattern of alternating potential influence onto the unit cells of the 18 × 18 1H‐TaS_2_ supercell, see Figure 5. For each unit cell where the influence of the MS moiré potential is considered, a constant energy offset of 136 meV has been added onto the on‐site terms of the downfolded Hamiltonian, which is in the typical order of magnitude for interlayer interactions in transition‐metal dichalcogenide based 2D materials [31].

### 4.7 Molecular Dynamics Simulations

A series of MD calculations were performed for 18 × 18 supercells of 1H‐TaS_2_ in order to model the thermodynamic stability of the CDW configuration. For temperatures of (20, 50, 100, 200) K, single‐ensemble NVT simulations were carried out using the i‐PI code [42, 43, 44]. The supercells were simulated over ∼7 ps to thermalize the system, using a simulation time step size of 4 fs. Canonical sampling was enforced by using the stochastic‐velocity‐rescaling thermostat method [45] with a relaxation time parameter of *τ* = 800 fs. NVT simulations of 70 replicas between *T* = 20 K and *T* = 200 K were performed using the replica‐exchange MD method [46] as implemented in i‐PI, with the simulation duration of the system spanning 1000 ps for the simulations in Figure 4 and Figure S15, and 500 ps for the additional simulations in Figures S13 and S14 (simulation time step size: 4 fs). To ensure canonical sampling, the PILE‐G thermostat method [47, 48] was used, with a relaxation time parameter of *τ* = 100 fs. We depict the averaged intensities at *T* = 20 K for comparison with experiment.

## Conflicts of Interest

The authors declare no conflicts of interest.

## Supporting information




**Supporting File**: adma74730‐sup‐0001‐SuppMat.docx.

## Data Availability

The data that support the findings of this study are available from the corresponding author upon reasonable request.
