# Supplementary material for: Moiré‐Induced Symmetry Breaking of Charge Order in van der Waals Heterostructures
Source: Adv Mater. 2026 Aug 28;38(59):e74730. doi: 10.1002/adma.74730 (PMC13689892; doi:10.1002/adma.74730)
Supplement: Supplementary file 1 [file ADMA-38-e74730-s001.docx]

Supplementary materials for

**Moiré-Induced Symmetry Breaking of Charge Order in van der Waals Heterostructures**

Sandra Sajan, Laura Pätzold, Tarushi Agarwal, Clara Pfister, Haojie Guo, Sisheng Duan, P. V. Sruthibhai, Mariana Rossi, Maria N. Gastiasoro, Sara Barja, Ravi P. Singh, Tim Wehling**^*^** and Miguel M. Ugeda**^*^**

******* *Corresponding authors:* [*tim.wehling@uni-hamburg.de*](mailto:tim.wehling@uni-hamburg.de) and [*mmugeda@dipc.org*](mailto:mmugeda@dipc.org)

# **Supplementary section 1. Mesoscopic characterization of bulk (PbS)_1.13_TaS_2_ and (SnS)_1.15_TaS_2_**

The X-ray diffraction (XRD) patterns, recorded at room temperature, of the grown single crystals of (PbS)_1.13_TaS_2_ and (SnS)_1.15_TaS_2_ are shown in Figures S1a and S1b, respectively. The corresponding optical image of the crystals are presented in the insets.

Bulk superconductivity in (PbS)_1.13_TaS_2_ was verified by the appearance of a diamagnetic response in temperature-dependent magnetization measurements carried out under zero-field-cooled warming (ZFCW) and field-cooled cooling (FCC) conditions at *H*^⟂^ = 1 mT, displayed in Figure S2a. The onset superconducting transition was measured at *T*_C_ ≃ 3.1 K.

Similarly, the bulk superconductivity in (SnS)_1.15_TaS_2_ was confirmed by diamagnetic signal in the temperature-dependent magnetization measurement, performed in zero field cooled warming (ZFCW) and field cooled cooling (FCC) mode at *H*^⟂^ = 1 mT, displayed in Figure S2b. The onset of the superconducting transition occurs at *T*_C_ ≃ 2.9 K. To determine the upper critical field (*H*^⟂^_C2_) value, temperature-dependent resistivity measurements were carried out under various applied magnetic fields oriented along the *c*-axis (*H* ‖ *c*), as shown in Figure S3a. The *H*^⟂^_C2_ value were estimated using the criterion *ρ* = 0.5·*ρ*_n_, where *ρ*_n_ indicates the normal state resistivity (at *T* = 4 K). For *H* ‖ *c* orientation, the obtained *T* dependence of *H*^⟂^_C2_ follows Ginzburg-Landau (GL) model, yielding *H*^⟂^_C2_ (0) ≃ 0.33 T, as shown in Figure S3b. This value is consistent with STM/STS data (see supplementary section 5).

**Figure S1.** Room temperature single-crystal x-ray diffraction patterns of **a**, (PbS)_1.13_TaS_2_ and **b**, (SnS)_1.15_TaS_2_. The diffraction peaks correspond exclusively to reflections from the basal (00*l*) planes, confirming the high crystallinity and preferred orientation of the layered crystals along the *c*-axis. Insets show optical microscopic images of the obtained single crystals.

**Figure S2.** Temperature-dependent magnetic susceptibility of **a** (PbS)_1.13_TaS_2_ and **b** (SnS)_1.15_TaS_2_, showing the onset of diamagnetic shielding associated with the superconducting transition in both systems.

**Figure S3.** **a**, Temperature-dependent electrical resistivity of (SnS)_1.15_TaS_2_ measured under different magnetic fields for *H*^||^*^c^* orientation (*H*^⟂^). **b**, *H*^⟂^_C2_ variation as a function of temperature. The dashed red line indicates the fitting with the GL model.

# **Supplementary section 2. Atomic scale morphology of the (SnS)_1.15_TaS_2_ compound**

**Figure S4. Atomic scale morphology of (SnS)_1.15_TaS_2_.** Atomically resolved STM images of **a**, the SnS layer (*V*_s_ = 1.2 V, *I* = 30 pA, *T* = 4.2 K) **b**, the 1H-TaS_2_ layer (*V*_s_ = 0.2 V, *I* = 0.2 nA, *T* = 3.8 K) and **c**, the 1H-TaS_2_ layer where the 1D is directly visible in the topography (*V*_s_ = -0.2 V, *I* = 0.15 nA, *T* = 3.8 K). The right part shows the inverted FFT of the image to guide the visualization of the moiré. **d** and **e**, Constant height nc-AFM images (2.2 nm × 2.2 nm) of the SnS and TaS_2_ layers, respectively (*T* = 3.8 K). Yellow (gray) balls correspond to Sn (S) atoms.

# **Supplementary section 3. Electronic structure of the (SnS)_1.15_TaS_2_ compound**

**Figure S5. Large-scale STS on the (SnS)_1.15_TaS_2_ compound.** Wide-bias d*I*/d*V* spectra acquired on TaS_2_ (orange) and PbS (red) layers (*T* = 3.8 K).

# **Supplementary section 4. Superconducting gap fitting of the monochalcogenide layers**

**Figure S6. BCS fit to the superconducting gap of the monochalcogenide layers.** Low-bias d*I*/d*V* spectra acquired on **a** PbS and **b** SnS measured at *T* = 0.34 K with the corresponding BCS fits.

# **Supplementary section 5. Magnetic field dependence of the superconducting gap of (PbS)_1.13_TaS_2_**

**Figure S7. Magnetic field dependence of the superconducting gap.** Low-bias d*I*/d*V* spectra sets acquired on **a** TaS_2_ and **b** PbS for different out-of-plane magnetic field values (*H*^⟂^) measured at
*T* = 0.34 K.

# **Supplementary section 6. Spatially resolved superconducting gap**

**Figure S8. Spatially resolved low-energy STS. a,** Low-bias d*I*/d*V* line spectra acquired on adjacent TaS_2_ (left) and PbS (right) terraces (*T* = 0.34 K). **b,** Corresponding 1D FFT.

# **Supplementary section 7. Broken lattice degeneracy in the CDW of (SnS)_1.15_TaS_2_ and electronic origin**

Figure S9a shows a large-scale STM image of the TaS_2_ layer in (SnS)_1.15_TaS_2_ with atomic resolution, whose FFT is shown in Figure S9b. As in (PbS)_1.13_TaS_2_, the FFT shows elongated CDW features ***q****_i_* along the Bragg directions with two distinct internal structures. The moiré peak is also visible at ***q***_moiré_ = (0.84 ± 0.01) Å^-1^$\cdot$***q****_x_*. The ***q***_1_ feature, orthogonal to ***q***_moiré_, exhibits intensity widely extended over Δ***q***_1,FWHM_ = (0.31 ± 0.10) Å ^-1^ around a maximum at $\frac{1}{2}$***Q***_B_ (2×2 in real space). The ***q***_2_ and ***q***_3_ features, rotated by 30° with respect to ***q***_moiré_, show equal intensities larger than that of *q_1_*, which span long arcs of Δ***q***_2,3,FWHM_ = (0.56 ± 0.12) Å ^-1^. Unlike ***q***_2_ and ***q***_3_ in (PbS)_1.13_TaS_2_, these features show a roughly constant intensity over their entire extent. As in (PbS)_1.13_TaS_2_, no intensity has been found in the ***q****_i_* features at $\frac{1}{3}$***Q***_B_ (3×3 in real space) for TaS_2_. These observations indicate that the threefold lattice degeneracy of the CDW in the 1H-TaS_2_ monolayer is also lifted in (SnS)_1.15_TaS_2_, as it is described for (PbS)_1.13_TaS_2_ in the main manuscript.

**Figure S9. CDW in the 1H-TaS_2_ layer of (SnS)_1.15_TaS_2_. a**, Large-scale STM image with atomic resolution in 1H-TaS_2_ (*V*_s_ = 0.08 V, *I* = 800 pA, *T* = 3.8 K) and **b**, its corresponding Fourier transform (FFT). **c**, Intensity profiles of the FFT along (0,0) and ***Q***_B,1_ (orange) and ***Q***_B,2_ (cyan).

To directly assess the extent to which the Pb- and Sn-based compounds exhibit quantitatively comparable CDW behavior, we summarize in Table S1 the experimental positions and widths of their Fourier features along with the experimental superconducting gaps for completeness. Both systems display the same qualitative lifting of the threefold CDW degeneracy, with ***q****_1_* distinct from the symmetry-related ***q****_2_* and ***q****_3_* components.

|  | **(PbS)_1.13_TaS_2_** | **(SnS)_1.15_TaS_2_** |
| --- | --- | --- |
| $\left\vert q_{moiré} \right\vert$ (Å⁻¹) | 0.84 ± 0.01 | 0.84 ± 0.01 |
| *q_1,max_* | *Q_B_*/2 | *Q_B_*/2 |
| *q_1_ width* (Å⁻¹) | 0.28 ± 0.08 | 0.31 ± 0.10 |
| *q_2,3,max_* | *3Q_B_*/8 and 5*Q_B_*/8 | Uniform intensity |
| *q_2,3_* width (Å⁻¹) | 0.57 ± 0.02 | 0.56 ± 0.12 |
| $\Delta_{TaS2}$(meV) | 0.38 ± 0.03 | 0.33 ± 0.03 |
| $\Delta_{MS}$(meV) | 0.35 ± 0.04 (PbS) | 0.260 ± 0.003 (SnS) |

**Table S1.** Quantitative comparison of the averaged CDW Fourier features and superconducting gaps in (PbS)_1.13_TaS_2_ and (SnS)_1.15_TaS_2_.

# **Electronic origin of the FFT features**

To further verify the electronic origin of the modulations, we acquired energy-resolved constant-height dI/dV maps in the TaS_2_ layer, such as that shown in Figure S10a, and measured over the same field of view of the topography image of Figure 3a. Their Fourier transforms display elongated qi features at the same wavevectors and with the same anisotropic structure as those observed in the topographic FFTs, showing that the modulations are also present in the local DOS. In addition, nc-AFM images acquired in comparable regions do not reveal any sizable structural corrugation or lattice distortion associated with the ***q***_i_ modulations. These observations support our assignment of the FFT features to electronic CDW modulations rather than to purely structural corrugation or topographic effects.

**Figure S10. CDW in the 1H-TaS_2_ layer of (SnS)_1.15_TaS_2_. a**, Upper panel, atomically resolved constant-height dI/dV map acquired in 1H-TaS_2_ (*V*_s_ = -0.05 V, *I* = 250 pA, *T* = 1.7 K) and its corresponding Fourier transform (FFT) below. **b** and **c**, Atomically resolved constant-height nc-AFM images of TaS_2_ layers and corresponding Fourier transform (FFT) below

# **Supplementary section 8: Downfolding model of undoped 1H-TaS_2_**

The results of the downfolding model relaxation for undoped monolayer 1H-TaS_2_ with a lattice constant of 3.34 Å are shown in Figure S11. The low-energy subspace consisting of three energy bands around the Fermi energy was transformed into a Wannier basis starting from orbitals of Ta *d_z_^2^*, *d_x_^2^_−y_^2^*, and *d_xy_* character. The top view of the 18×18 supercell in Figure S11a indicates the relaxation into the native 3×3 CDW reconstruction via arrows, rescaled by a factor of 20 for better visibility. The distortion is limited to the *xy* direction, with the *z* direction being undistorted, see the side view in Figure S11a. The reconstruction into the CDW is accompanied by an asymmetric double-well potential in the total energy, as seen in Figure S11 b. The global minimum located at a displacement of *α* = 1 corresponds to the CDW distortion shown, with *α* = 0 being the structure in the symmetric phase and the displacement being linearly interpolated in-between. By relaxing into the CDW, an energy of about 750 meV is gained in the 18×18 supercell, which equals a normalized binding energy of about 2.3 meV per unit cell.

To visualize the phonon contribution responsible for the CDW instability, we calculate the static structure factor

$$S\left( \boldsymbol{q} \right)=\frac{1}{N_{\mathrm{at}}^{2}}\left| \sum_{l=1}^{N_{\mathrm{at}}} e^{-i\boldsymbol{q}\cdot\boldsymbol{R}_{l}} \right|^{2}$$

see Figure S11c. It represents the Fourier transform of the relaxed 3×3 atomic superstructure in Figure S11a, while also providing an estimation of the momentum-dependent scattering intensity measured in experiment. The structure factor is peaked in reciprocal space around $\frac{2}{3}\Gamma M$ ($\frac{1}{3}$***Q***_B_), which corresponds to the expected 3×3 CDW reconstruction. The less intense peak at ***q*** = $K$ is also characteristically visible for the 3×3 CDW. A closer look at the corresponding DFPT phonon dispersion of the 1H-TaS_2_ unit cell in Figure S11d also verifies the position of the expected soft phonon mode at $\frac{2}{3}\Gamma M$ ($\frac{1}{3}$***Q***_B_) for the 3×3 superstructure. While no phonon instability can be found at the K point, the corresponding displacements of √3×√3 are commensurate with the 3×3 CDW and couple anharmonically to the unstable modes at $\frac{2}{3}\Gamma M$ .

The band structure of the CDW phase (orange) in undoped 1H-TaS_2_ is shown in Figure S11e, with the weights of the unfolded bands visualized via logarithmic color bar and marker size. The bands and the Fermi level of the symmetric phase are shown in blue. The band structure of the CDW phase is unfolded from the supercell to the unit cell, and the Fermi level (indicated via dashed lines) stays nearly constant between the symmetric phase and the CDW. Additionally, new band contributions located at $\frac{2}{3}\Gamma M$ are found in the band structure of the CDW phase, in line with the wavevector of the soft phonon mode found in the phonon dispersion in Figure S11d.

**Figure S11. Model relaxation of undoped 1H-TaS_2_.** 3×3 CDW in undoped monolayer 1H-TaS_2_. **a**, Top and side view of the relaxed crystal structure from model calculations performed on an 18×18 supercell with a lattice constant of 3.34 Å. The relaxation into the 3×3 charge density wave configuration is indicated via arrows that are rescaled by a factor of 20. **b**, Total free energy as function of the displacement α for the 3×3 CDW shown in **a**. The relaxation started from the symmetric phase (SYM, *α* = 0), revealing a minimum at α = 1 corresponding to the CDW distortion. The resulting total energy gain of about 750 meV represents the formation energy of the CDW. **c**, Structure factor *S*(***q***) of the relaxed structure in **a**, with the intensity peaks at $\frac{2}{3}\Gamma M$ of the phononic Brillouin zone indicating the 3×3 CDW. A less intense contribution at the $K$ and $K’$ points, also characteristic for the 3×3 CDW, can be seen. **d**, Phonon dispersion of 1H-TaS_2_ obtained via DFPT. The soft mode at $\frac{2}{3}\Gamma M$ is indicative of the 3×3 CDW. **e**, Reconstructed band structure of the low-energy subspace as obtained by DFT (blue) and via model relaxation into the CDW structure (orange). The logarithmic color bar (right) as well as the size of the markers are used to visualize the weights of the unfolded bands. The relaxed band structure of the CDW has been unfolded from the supercell to the unit cell, revealing several replica bands, associated with the symmetry breaking of the CDW phase. The nearly identical Fermi energies of both band structures are indicated via dashed lines in blue and orange, respectively.

# **Supplementary section 9: Influence of electron doping on CDW symmetry in 1H-TaS_2_**

Analyzing the influence of electron doping on the 1H-TaS_2_ monolayer in our downfolding model reveals a variety of possible CDW symmetries that our model relaxes into for different doping levels of the unit cell. The most commonly appearing CDW configurations were the commensurate 3×3 CDW for the undoped system and the commensurate 2×2 CDW for higher electron doping, with an incommensurate CDW (ICCDW, wavevector of $\frac{8}{9}\Gamma M$, ~2.25×2.25) in direct energetic competition.

Via the difference in corresponding binding energies between the energetically most favorable and the second most favorable (IC)CDW, we visualize the occurrence of different configurations as a function of the doping and the electronic smearing in a CDW phase diagram in Figure S12. For each doping, an *ab-initio*-based model was set up, with the binding energies of each (IC)CDW being calculated via enforcing the input structure for model calculations at values of different doping and smearing (see black circles). The rest of the energy landscape is interpolated between these values. For each (IC)CDW symmetry, the regions where it is the energetic ground state are indicated via the shading of the colorbars, with different (IC)CDW symmetries being energetically favorable for different doping levels. Note that the colorbars on the right are scaled for best visibility and do not share a unified maximum. The undoped system favors the 3×3 symmetry (pink), with higher electron doping it undergoes a shift towards the 2×2 CDW as the energetic ground state (orange). For even higher electron doping, the 2×2 CDW competes with the 2×2-like ICCDW (green), which becomes slightly favorable. Between these regions, white phase boundaries are visible where both (or multiple) CDW configurations show identical binding energies. For higher electronic smearing (above ≈60 meV) as well as for higher doping (*x* > 0.25), no CDW configuration is found to be stable. We therefore located a region in the phase diagram where dominant 2×2 contributions are expected for an electron doping of approximately *x* = 0.1 to *x* = 0.2, therefore doping shifts the dominant structure factor contribution towards the $M$ point.

**Figure S12. CDW phase diagram of electron-doped 1H-TaS_2_.** Phase diagram of monolayer 1H-TaS_2_ as a function of the electronic smearing *k*_B_*T* and the electron doping *x*. The binding energies of each (IC)CDW were calculated via enforcing the input structure for model calculations at different doping and smearing levels (black circles), with the rest of the energy landscape being interpolated between. The differently colored regions show the expected (IC)CDW ground state by indicating the energy difference between the most favorable and the second most favorable configuration. The 3×3 CDW shown in pink is dominant for the undoped system, while the 2×2 CDW (orange) equals the ground state for an electron doping of *x* = 0.1 - 0.2. For higher doping, an ICCDW with a wavevector of $\frac{8}{9}\Gamma M$ (green) emerges. Note that the colorbars on the right are scaled for best visibility. Between these regions, white phase boundaries are visible where both (or multiple) CDW configurations show identical binding energies. For electronic smearing above 60 meV as well as for doping above *x* = 0.25, no CDW configuration is found to be stable.

Building on these results, we use MD calculations to explore the sensitivity of the CDW wave vector to the doping level. Figure S13 shows the structure factors of the resulting favored structures for an 18x18 supercell of 1H-TaS_2_ at *T* = 20 K. For the undoped case, the 3x3 CDW dominates as expected, while for an intermediate doping of *x* = 0.1 to *x* = 0.15, the shift towards the Brillouin zone edge as well as competition between the different CDWs is visible via coexisting contributions, with additional slightly smeared-out characteristics along $\Gamma M$. For higher doping (*x* = 0.2), the 2x2 as well as the 2x2-like ICCDW are in competition due to their close ground state energies predicted by our model calculations in Figure S12. For the Pb-based misfit layered compound, the doping level of *x* = 0.2 was estimated via ARPES results^1^ by determining the change in the Fermi area compared to an isolated monolayer. Thus, a doping of *x* = 0.2 was chosen for the qualitative simulation of the misfit layered compound in our model. As constructed, this model is geared towards the Pb-based misfit compounds.

**Figure S13. Doping dependence of the CDW wave vector in MD calculations of 1H-TaS_2_.** Structure factors of favored relaxed structures of an 18x18 supercell of monolayer 1H-TaS_2_ obtained via MD calculations, shown for *T* = 20 K. For the undoped case, the 3x3 CDW dominates with a contribution located at $\frac{2}{3}\Gamma M$. For an intermediate doping of *x* = 0.1 to *x* = 0.15, the system shifts into the 2x2 symmetry, shown via dominant contributions at $M$. Additionally, coexisting and smeared-out contributions show the energetic competition between different (IC)CDW configurations. For the highest doping shown (*x* = 0.2), the commensurate 2x2 CDW competes with a 2x2-like ICCDW as the energetic ground state.

# **Supplementary section 10: Influence of moiré potential amplitude modulation on CDW symmetry in 1H-TaS_2_**

The strength of the moiré potential modulation amplitude added as an energy offset onto the on-site terms of the downfolded Hamiltonian is treated as an adjustable parameter to account for the interlayer hybridization with the underlying square lattice, see Methods section and Figure 5 of the main manuscript for more details. This hybridization breaks the threefold symmetry of the structure factor. The sensitivity of the observed symmetry breaking to moiré-potential modulation amplitude is demonstrated in Figure S14 for the undoped 18x18 supercell at *T* = 20 K. For the system without moiré potential, the expected 3x3 symmetry is shown. By increasing the amplitude of the vertically striped external moiré potential, a smeared-out moiré contribution located along $\Gamma K$ emerges. Perpendicular to that direction, a preferred axis along $\Gamma M’$ manifests for larger moiré amplitudes, where the intensity of the corresponding structure factor contribution is enhanced. Thus, the former threefold symmetry of the three $\Gamma M$ directions is broken due to the applied moiré potential. Typical interlayer interactions in TMD-based 2D materials are on the order of ~100 meV^2^ We use the case of 136 meV, which is on this order, as a qualitative account for the potential modulation in the misfit compounds.

**Figure S14. Dependence of the CDW wavevector on the moiré amplitude in MD calculations of 1H-TaS_2_.** Structure factors of favored relaxed structures of an 18x18 supercell of monolayer 1H-TaS_2_ obtained via MD calculations, shown for *T* = 20 K. Without external moiré potential, the system exhibits the expected 3x3 CDW. With increasing potential amplitude, a smeared contribution along $\Gamma K$ emerges, corresponding to the vertically striped moiré potential. Perpendicular to that, a preferred axis along $\Gamma M’$ manifests as an enhanced intensity of the structure factor contribution at $\frac{2}{3}\Gamma M’$, breaking the former three-fold symmetry of the CDW wavevectors.

# **Supplementary section 11: Temperature dependence of the symmetry-broken CDW state in the misfit layered compound**

To analyze the temperature-dependence of the symmetry-broken CDW state by means of MD calculations (*x* = 0.2, moiré modulation of 136 meV), we show the structure factors associated with different replicas at different temperatures from *T* = 20 K to *T* = 200 K in Figure S15. The qualitative characteristics of the structure factor appear largely stable up to *T* = 50 K, with some signatures of thermal broadening and enhanced diffuse scattering. In the temperature range of *T* = 100 K to *T* = 200 K, the CDW order fades out, signaling the phase transition into the high-temperature non-symmetry broken phase. The moiré contributions along $\Gamma K$ remain stable even for the highest replica temperature of *T* = 200 K.

**Figure S15. Temperature dependence of symmetry-broken CDW state in MD calculations of misfit layered compound.** Structure factors of favored relaxed structures of an 18x18 supercell of monolayer 1H-TaS_2_ via MD calculations, shown for *x* = 0.2 and a moiré potential of 136 meV at temperatures between *T* = 20 K and *T* = 200 K. The qualitative characteristics of the symmetry-broken structure factor are thermally stable for temperatures up to *T* = 50 K. For higher temperatures (between *T* = 100 K and *T* = 200 K) the CDW order vanishes, while the moiré contribution along ΓK remains stable up to *T* = 200 K.

# **Supplementary section 12: Fourier analysis**

In Figure S16, we show the effect of the mask shape, apodization, and size on the inverse Fourier-transform reconstruction. The resulting real-space spatial distribution and characteristic dimensions of the CDW regions remain essentially unchanged for hard (rectangular, elliptical), and apodized (multi-elliptical, Hann, and Blackman–Harris) masks.

**Figure S16. Inverse Fourier analysis and masks.** Upper row, topography and corresponding FFT of the region boxed in figure 3a. The middle column from the second row show different masks applied to the *q_1_* feature in the FFT to generate the corresponding inverse FFT maps (right column).

**References**

1.	K. P., S. *et al.* Ising superconductivity in the bulk incommensurate layered material (PbS)_1.13_(TaS_2_). *Phys. Rev. B* **111**, 054509 (2025).

2.	Kuc, A., Zibouche, N. & Heine, T. Influence of quantum confinement on the electronic structure of the transition metal sulfide TS_2_. *Phys. Rev. B* **83**, 245213 (2011).
